# Synthetic Biology and Systems Metabolic Engineering of Aminoglycoside Antibiotics: From Biosynthetic Pathways to Resistance-Evading and Low-Toxicity Design

**DOI:** 10.3390/microorganisms14102209

**Published:** 2026-10-01

**Authors:** Shan Chu, Yuqing Chu, Mohan Huang, Haoran Lu, Zhiqiao Liu, Shuo Wang, Hongquan Zhao, Xiaotang Chen

**Affiliations:** College of Medical Engineering, Jining Medical University, Jining 272067, China; chushan@stu.mail.jnmc.edu.cn (S.C.); chuyuqing@stu.mail.jnmc.edu.cn (Y.C.); huangmohan@stu.mail.jnmc.edu.cn (M.H.); luhaoran@stu.mail.jnmc.edu.cn (H.L.); liuzhiqiao@stu.mail.jnmc.edu.cn (Z.L.); wangshuo@stu.mail.jnmc.edu.cn (S.W.); zhaohongquan@stu.mail.jnmc.edu.cn (H.Z.)

**Keywords:** aminoglycoside antibiotics, biosynthetic pathways, synthetic biology engineering, antimicrobial resistance, toxicity optimization, systems metabolic engineering

## Abstract

Aminoglycoside antibiotics are important therapeutic agents in the clinical treatment of Gram-negative bacterial infections; however, the spread of antimicrobial resistance, together with dose-limiting ototoxicity and nephrotoxicity side effects, substantially restricts their clinical utility. Recent advances in synthetic biology and systems metabolic engineering have provided powerful approaches for elucidating and engineering the biosynthetic pathways of these antibiotics. This review systematically summarizes the conserved biosynthetic logic of aminoglycosides, with particular emphasis on the 2-deoxystreptamine and streptidine core scaffold assembly and on the catalytic features and substrate selectivity of four major enzyme families involved in aminoglycoside biosynthesis: glycosyltransferases, pyridoxal phosphate-dependent aminotransferases, S-adenosyl-L-methionine-dependent methyltransferases, and phosphorylation/deamination enzymes. On this basis, the core strategies for synthetic biology and systems metabolic engineering of aminoglycoside antibiotics are reviewed from five perspectives: genetic circuit design, chassis cell optimization, cell-free synthesis, heterologous de novo biosynthesis, and systems biology-driven global optimization. Using gentamicin, kanamycin, tobramycin, apramycin, neomycin, and streptomycin as representative examples, we highlight the distinctive features of their respective biosynthetic pathways and discuss their potential for synthetic biology-based exploitation. Particular attention is given to the development of novel derivatives, including resistance-evading derivatives such as plazomicin, low-toxicity designs based on 4-monosubstituted apramycin structures, and advances in combinatorial biosynthesis for generating hybrid antibiotics. Finally, key challenges are examined, including heterologous expression compatibility, bottlenecks in product yield, and an incomplete understanding of structure–activity relationships. Emerging strategies for overcoming these challenges are also discussed, with a focus on cutting-edge technologies such as artificial intelligence-driven dynamic regulation, genome-scale metabolic model-guided design, and cell-free synthesis systems.

## 1. Introduction

Aminoglycoside antibiotics (AGs) are composed of amino sugars and aminocyclitols linked by glycosidic bonds. Following the discovery of streptomycin in 1943, one of its landmark early clinical applications was the treatment of *Mycobacterium tuberculosis* infection. AGs have since become important therapeutic agents for the treatment of severe Gram-negative bacterial infections [1,2]. AGs exert their antibacterial activity primarily by binding to the 30S subunit of the bacterial ribosome, thereby disrupting protein synthesis and producing a rapid bactericidal effect [3]. Due to their broad antibacterial spectrum, rapid bactericidal activity, and potent synergistic effects with other antibiotics, AGs remain important agents for the treatment of serious bacterial infections [4]. However, prolonged and widespread antibiotic use has contributed to the increasingly serious problem of bacterial antimicrobial resistance. The major mechanisms of aminoglycoside resistance include drug inactivation mediated by aminoglycoside-modifying enzymes (AMEs), ribosomal target site modifications, and efflux pump overexpression, among which AME-mediated drug inactivation is the most prevalent [5,6]. The global dissemination of antibiotic resistance genes, particularly the emergence of high-level resistance mediated by 16S rRNA methyltransferases (RMTases) [7], has further complicated the clinical management of bacterial infections. In addition, ototoxicity and nephrotoxicity associated with AGs substantially limit AG’s clinical utility [8].

The structural complexity of natural products has hindered the development of novel AGs by chemical semisynthesis, as multistep protection-deprotection strategies are often cumbersome and result in low overall yields, making it challenging to overcome the structural limitations of existing compounds [9,10]. The emergence of synthetic biology has provided a transformative approach to this challenge. By reconstructing and optimizing biosynthetic pathways in microbial hosts, complex natural products and their derivatives can be synthesized with greater efficiency and selectivity while reducing reliance on harsh chemical processes and enabling more sustainable production [11,12]. By incorporating systems biology, particularly genome-scale metabolic models (GSMMs) and metabolic flux analysis, researchers can elucidate and optimize the metabolic networks of host cells from a global perspective, providing quantitative guidance for synthetic biology-based engineering [13]. In recent years, artificial intelligence (AI)-based dynamic regulation and multi-omics integration have further strengthened the convergence of systems biotechnology and synthetic biology, ushering new “smart cell factories” for AGs.

This review aims to provide a reference for biosynthetic research and structural optimization of AGs by systematically examining five aspects: (1) their conserved biosynthetic pathways, (2) core synthetic biology-based engineering strategies, (3) the exploitation of pathway-specific features in representative antibiotics, (4) advances in the development of novel derivatives, and (5) current challenges and future prospects. Rather than focusing exclusively on biosynthetic mechanisms or engineering strategies, this review seeks to bridge these two areas and, more importantly, demonstrate how biosynthetic knowledge can be translated into the rational design and efficient production of next-generation AGs with reduced resistance and toxicity (Table 1).

## 2. Conserved Biosynthetic Pathways of AGs

Since the discovery of streptomycin in 1943 [1], the biosynthetic pathways of AGs have been extensively and continuously investigated. With the rapid development of genome sequencing technologies and breakthroughs in genetic manipulation systems since the beginning of the 21st century, substantial progress has been made in understanding the biosynthetic mechanisms of AGs. AGs are predominantly biosynthesized in actinomycetes [1,16], including *Streptomyces* and *Micromonospora*. Their biosynthesis generally involves the formation of characteristic aminocyclitol scaffolds, followed by glycosylation and other enzymatic tailoring reactions that generate the final pseudo-oligosaccharide structures.

### 2.1. Biosynthesis of Core Scaffold Precursors

Aminocyclitol scaffolds form the structural foundation of AGs. They can be divided into two major categories according to their origins: those derived from 2-deoxy-scyllo-inosose (2-DOI), primarily found in antibiotics containing 2-deoxystreptamine (2-DOS), and those derived from myo-inositol-1-phosphate (MIP), found in antibiotics such as streptomycin and spectinomycin [16].

#### 2.1.1. Biosynthetic Pathway of 2-DOS

**Figure 1:**
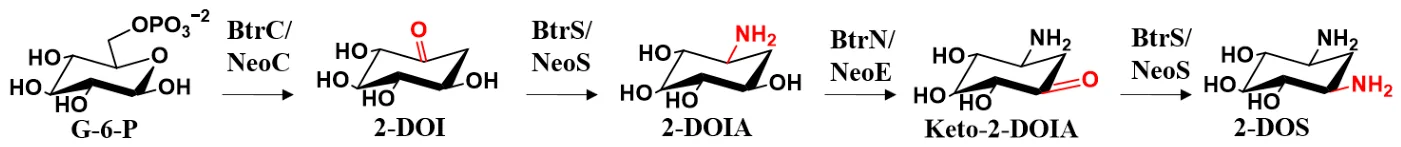
Biosynthetic pathway of 2-DOS. Chemical structures were drawn using ChemDraw 22.0 (PerkinElmer, Waltham, MA, USA).

The most clinically important AGs, including neomycin, kanamycin, and gentamicin, all have 2-DOS as their primary aminocyclitol scaffold. The biosynthetic pathway of 2-DOS has been fully elucidated through in vitro enzymatic studies [17] (Figure 1). It begins with the conversion of D-glucose-6-phosphate (G-6-P) to 2-DOI, catalyzed by a 2-DOI synthase (e.g., BtrC in butirosin biosynthesis and NeoC in neomycin biosynthesis) [17]. Although 2-DOI synthase is part of the 3-dehydroquinate (DHQ) synthase family, its catalytic mechanism differs significantly. Kakinuma et al. [2] elucidated the bioconversion pathway from glucose to 2-DOI based on deuterated glucose feeding experiments. A crystal structure analysis of BtrC revealed an active site structurally similar to that of DHQ synthase, but with distinct substrate and reaction specificities arising from differences in key amino acid residues. Nango et al. [18] subsequently determined the crystal structure of BtrC in complex with the substrate analog inhibitor carba-G-6-P, NAD^+^, and Co^2+^, providing insights into both mechanistic similarities and functional differences between 2-DOI synthase and DHQ synthase in primary metabolism.

After 2-DOI is generated, it undergoes two aminotransferase reactions and one dehydrogenation reaction to be converted into 2-DOS. First, an L-glutamine:2-DOI aminotransferase (e.g., BtrS/NeoS) catalyzes the conversion of 2-DOI to 2-deoxy-scyllo-inosamine (2-DOIA) [1,2]. Then, 2-DOIA is oxidized by a dehydrogenase to generate keto-2-DOIA. Notably, there are two distinct mechanisms that catalyze this dehydrogenation reaction. In neomycin biosynthesis, the NAD^+^-dependent dehydrogenase NeoE catalyzes the reaction, whereas in butirosin biosynthesis, the unique radical S-adenosyl-L-methionine (SAM) dehydrogenase BtrN is responsible [1]. BtrN was the first radical SAM enzyme discovered to catalyze the anaerobic oxidation of a hydroxyl group [19]. In the final step, the aminotransferase BtrS/NeoS once more catalyzes the transamination of keto-2-DOIA to generate 2-DOS.

#### 2.1.2. Biosynthesis of Streptidine

The core aminocyclitol of streptomycin is streptidine. There are three key differences between the 2-DOS and streptidine pathways: the initial precursor of the streptidine pathway is inositol rather than G-6-P; it incorporates a unique amidination step; and its glycosyl components differ substantially from those of 2-DOS-containing antibiotics [16]. This pathway proceeds through a series of enzymatic modifications, including inositol oxidation, aminotransfer, phosphorylation, and amidination, ultimately forming the streptidine core. Streptidine is then sequentially glycosylated with streptose and *N*-methyl-L-glucosamine to complete streptomycin biosynthesis (see Section 4.6 for a detailed description of the streptomycin biosynthetic pathway) [20,21,22].

#### 2.1.3. Biosynthesis and Supply Mechanisms of Common Glycosyl Donors

Glycosylation modifications of AGs require activated nucleoside diphosphate (NDP)-sugars as glycosyl donors [9]. Antibiotics containing 2-DOS (e.g., kanamycin and gentamicin) primarily use uridine diphosphate (UDP)-activated sugars (e.g., UDP-glucose and UDP-xylose), while streptomycin and related compounds use TDP-activated deoxysugar pathways. Thymidine diphosphate (TDP)-glucose, the glycosyl donor in streptomycin biosynthesis, is generated from glucose-1-phosphate and deoxythymidine triphosphate (dTTP) in a pyrophosphorylase-catalyzed reaction [23]. UDP-xylose donates a xylose moiety in the biosynthesis of certain aminoglycosides, such as gentamicin [24]. NDP-heptose synthesis (used to provide the heptose moiety in hygromycin B) is more complex with multiple steps, including sedoheptulose-7-phosphate isomerization, phosphorylation, and NDP activation [9]. The types of glycosyl donors determine the diversity of AG glycosylation modifications.

### 2.2. Key Modifying Enzyme Families and Their Catalytic Mechanisms

**Figure 2:**
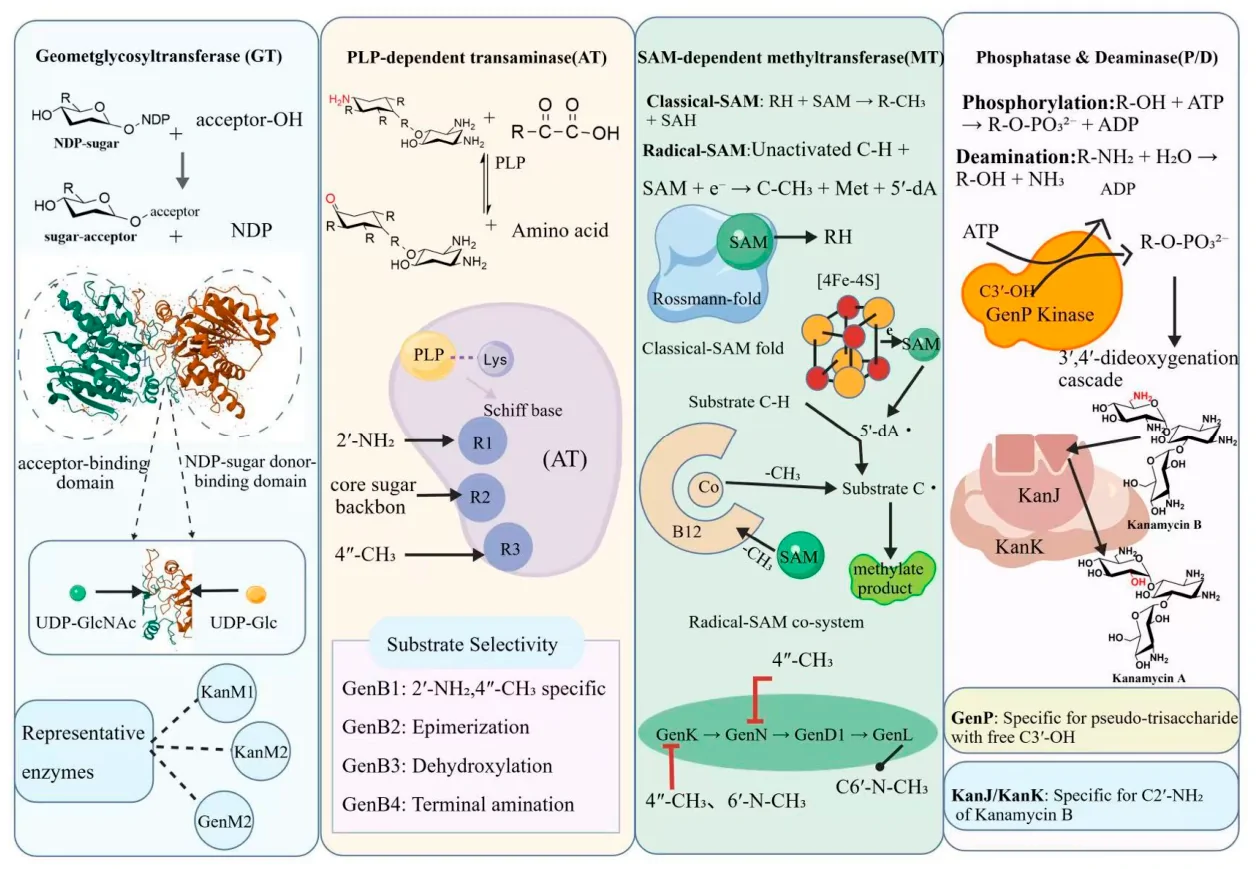
Key modifying enzyme families and their catalytic mechanisms. Chemical structures were drawn using ChemDraw.

The four major families of modifying enzymes involved in AG biosynthesis (glycosyltransferases, pyridoxal phosphate (PLP)-dependent aminotransferases, SAM-dependent methyltransferases, and phosphorylation/deamination enzymes) are highly conserved across various aminoglycosides and constitute a common enzymatic toolbox for synthetic biology-based engineering (Figure 2) [11,14]. However, the substrate specificity and selectivity rules governing these enzyme families remain only partially understood, and general principles that can directly guide rational design have yet to be established. Elucidating these rules therefore represents a central challenge for the synthetic biology-based engineering of AG biosynthesis.

#### 2.2.1. Glycosyltransferase Systems

Glycosyltransferases, which attach activated NDP-sugars to the aminocyclitol scaffold or extend existing sugar chains, are the most critical structural determinants in AG biosynthesis [17]. Based on their substrate specificity, glycosyltransferases can be categorized as substrate-specific or substrate-promiscuous. For example, in kanamycin biosynthesis, the substrate-flexible glycosyltransferase KanM1 uses UDP-*N*-acetylglucosamine as the donor to produce paromamine and UDP-glucose as the donor to produce 2′-deamino-2′-hydroxyparomamine [11,25]. KanM2 has even greater substrate promiscuity, recognizing multiple pseudodisaccharides as glycosyl acceptors and utilizing UDP-glucose or UDP-kanosamine as glycosyl donors [9,26]. This broad substrate flexibility is the molecular basis of glycosyltransferases as “exchangeable modules” and provides enzymatic tools for glycosylation-based structural modification. However, different glycosyltransferases have widely varying catalytic efficiencies with non-natural donors. The determinants of active-site “plasticity” are incompletely understood, which limits the ability to rationally design highly compatible enzyme variants. Although advances have been made in domain swapping and high-throughput screening, the efficiency of high-performance chimeric enzymes generation requires further improvement.

#### 2.2.2. PLP-Dependent Aminotransferase Systems

PLP-dependent aminotransferases are among the core catalytic elements responsible for the structural diversity of AGs. These enzymes convert hydroxyl groups into amino groups, a key modification that determines the antibacterial activity of AGs. The catalytic mechanism of PLP-dependent aminotransferases follows the classical pattern of PLP-dependent transaminases, with substrate selectivity determined by the subsite architecture of the active center. For example, the biosynthetic gene cluster (BGC) of gentamicin encodes four PLP-dependent aminotransferases, GenB1, GenB2, GenB3, and GenB4, each with distinct substrate specificity and catalytic function [14]. The crystal structure of GenB1 revealed that the active center consists of three subsites, R1, R2, and R3, which collectively determine the strong preference of GenB1 for substrates bearing a 2′-amino group and a 4″-methyl group [27,28]. In addition to aminotransferase activity, GenB2 also acts as an epimerase by catalyzing the interconversion between JI-20Ba and JI-20B and between gentamicin C2a and C2 [29]. GenB3 exerts didehydroxylation activity, catalyzing the dephosphorylation at C-3′and dehydration at C-4′,5′of phosphorylated JI-20A [30]. The subsite structures and key residues are conserved among the different PLP-dependent aminotransferases and underlie the structural basis of the substrate recognition and functional differentiation of this enzyme class. However, general rules for how to expand the substrate scope while retaining amination efficiency to achieve selective modifications at non-natural sites remain lacking.

#### 2.2.3. SAM-Dependent Methyltransferase Systems

Methylation is an important mechanism for structural diversification in AGs. Based on their catalytic mechanisms, AG methyltransferases can be broadly classified into two classes: classical SAM-dependent *N*-methyltransferases (e.g., GenN and GenL) and unique cobalamin-dependent radical SAM methyltransferases (e.g., GenD1 and GenK) [14,31]. These two classes employ fundamentally different catalytic strategies. SAM-dependent enzymes transfer methyl groups through nucleophilic substitution, whereas cobalamin-dependent enzymes use [4Fe-4S] clusters and vitamin B12 as cofactors to catalyze radical-mediated methylation at otherwise inactivated carbon centers [14,31]. In gentamicin biosynthesis, four methyltransferases cooperate to catalyze sequential methylation at the C-6′, N-3″, and C-4″ positions [14,27]. The regulatory logic of this pathway is reflected in the distinct substrate selectivity of each enzyme. GenK acts on all pseudotrisaccharide intermediates but is inhibited by the presence of 4″-methyl and 6′-*N*-methyl groups; GenN activity is inhibited by 4″-methyl groups; GenD1 activity is largely unaffected by other methyl groups; and GenL only catalyzes the 6′-*N*-methylation of C2 and C1a [27,31]. This relatively stringent selectivity ensures directed metabolic flux in wild-type strains and provides the molecular basis for metabolic branch blocking and targeted methylation engineering. However, single-point mutations are often insufficient to fully alleviate substrate inhibition, highlighting the need for combinatorial mutation strategies to achieve effective pathway remodeling.

#### 2.2.4. Phosphorylation and Deamination Systems

Phosphorylation and deamination reactions synergistically support structural optimization and product conversion of AGs. Phosphorylation is often the initiating step in deoxygenation reactions. For example, the 3′-*O*-phosphotransferase GenP catalyzes the phosphorylation of C-3′-OH, which undergoes 3′,4′-didehydroxylation to generate products such as sisomicin [32]. Deamination reactions mediate the interconversion between products. For example, the KanJ–KanK system catalyzes deamination at C-2′ to convert kanamycin B to kanamycin A [9,11], and the homologous system GenJ–GenK2 is a key participant in gentamicin B synthesis [28]. These two classes of enzyme systems generally exhibit high substrate specificity. When constructing heterologous pathways by replacing homologous enzymes, recognition requirements for specific intermediates must be carefully matched; otherwise, the metabolic flux is likely to be interrupted.

## 3. Core Strategies in Synthetic Biology and Systems Metabolic Engineering

**Figure 3:**
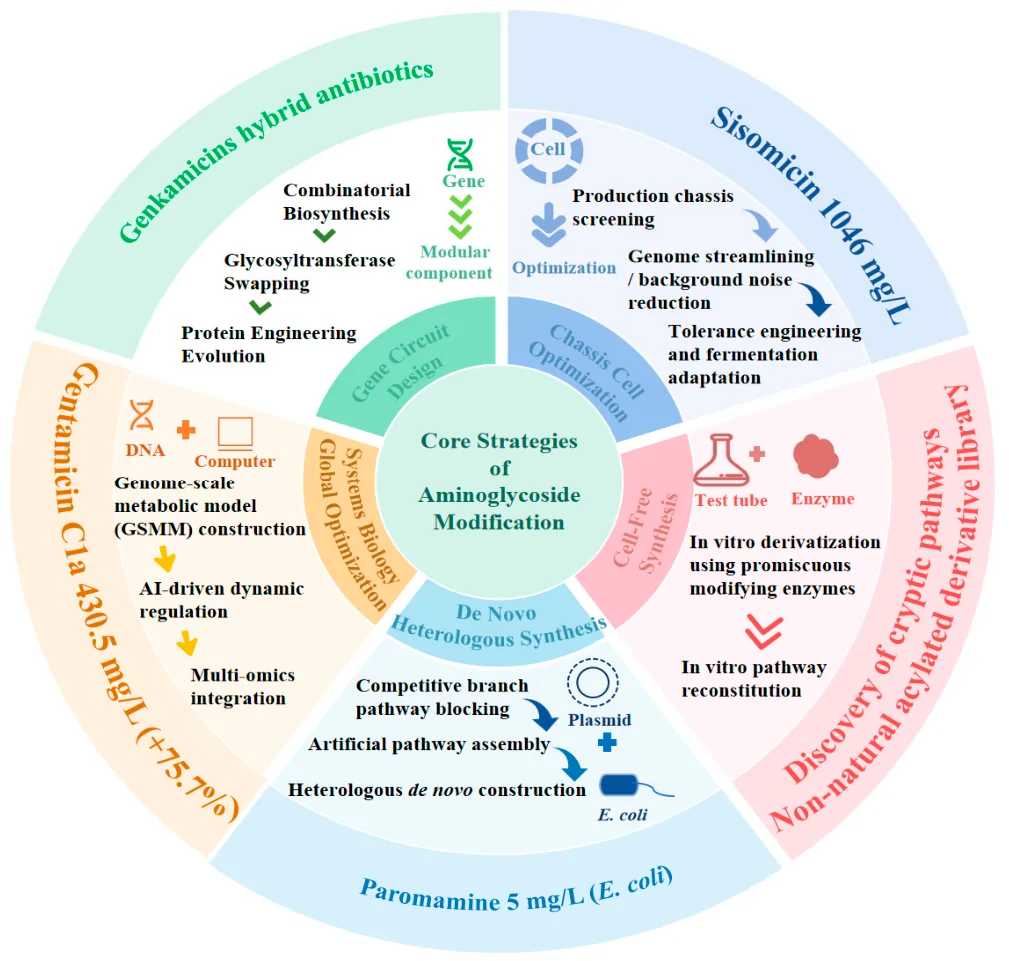
Core strategies in synthetic biology and systems metabolic engineering.

As bacterial resistance continues to intensify, the inherent limitations of traditional chemical semisynthesis in the development of novel AGs have become increasingly apparent [9]. The rapid emergence of synthetic biology has opened up new avenues for optimizing existing AGs and generating novel derivatives [11]. This section systematically examines the applications and advances of synthetic biology and systems biotechnology in AG research and development from five perspectives: genetic circuit design, chassis cell optimization, cell-free synthesis, heterologous de novo biosynthesis, and systems biology-driven global optimization (Figure 3).

### 3.1. Genetic Circuit Design

The BGC of AGs, which is the genetic basis for their natural synthesis, contains the complete genetic information responsible for molecular scaffold construction, group modifications, product transport, and host self-resistance [17]. A core synthetic biology approach is the rational design and engineering of the minimal functional units of biosynthesis, of both enzymes and gene modules, by introducing heterologous genes or directionally modifying endogenous genes to reprogram microbial metabolic pathways and thereby synthesizing structurally novel non-natural aminoglycoside derivatives [12].

#### 3.1.1. Combinatorial Biosynthesis and Side-Chain Module Assembly

The 4-amino-2-hydroxybutyric acid (AHBA) side chain modification is a key step in the synthesis of second-generation semisynthetic antibiotics, such as amikacin [32]. The natural biosynthetic mechanism that generates this side chain originates from the butirosin biosynthetic system. Researchers have successfully cloned and characterized seven key genes (*btrI*, *btrJ*, *btrK*, *btrO*, *btrV*, *btrH*, and *btrG*) from the butirosin BGC responsible for the biosynthesis and loading of the AHBA side chain [33]. The introduction of this gene set as a “side-chain installation module” into a kanamycin-producing strain and heterologous hosts such as *Streptomyces venezuelae* generates a specific modification at the *N*-1 position of kanamycin A and kanamycin X, producing amikacin and 1-*N*-AHBA-kanamycin X with excellent activity against resistant bacteria [26,33]. The findings of this study confirmed the practical value of combinatorial biosynthesis for the directed biosynthetic engineering of resistance-evading AGs. However, this type of modular replacement is often constrained by the steric limitations of substrate recognition pockets and interference from host modifying enzymes. Therefore, the loading efficiency may be significantly reduced in non-natural hosts.

#### 3.1.2. Glycosyltransferase Exchange and the Creation of Hybrid Antibiotics

Modifications of sugar moieties represent another important strategy for expanding AG structural diversity. As key pharmacophores of aminoglycosides, sugar moieties strongly influence both the pharmacological activity and toxicity of these compounds, depending on their composition and linkage patterns. The substrate specificity of glycosyltransferases is a major determinant of glycosylation-pattern diversity and can be deliberately altered through enzyme exchange. For example, by exploiting the substrate promiscuity of KanM2, a glycosyltransferase from the kanamycin biosynthetic pathway, researchers introduced KanM2 as a sugar-replacement module into *Micromonospora echinospora*, the natural producer of gentamicin [34]. In vivo heterologous replacement revealed that the endogenous glycosyltransferase GenM2 was replaced by the exogenous KanM2 to use glucose instead of the natural xylose as the glycosyl donor in gentamicin biosynthesis. This replaced the purpurosamine moiety in gentamicin with kanosamine and created a series of structurally novel “gentamicin–kanamycin” hybrid aminoglycosides (genkamicins) [35]. Structure–activity relationship (SAR) and toxicity evaluation studies of representative hybrid derivatives such as genkamicin C2a (GK-C2a) revealed that these derivatives retained potent antibacterial activity against resistant bacteria with markedly reduced ototoxicity and nephrotoxicity [35]. In an in vivo zebrafish toxicity model, toxicity levels of GK-C2a were significantly lower than those of clinically used aminoglycoside reference drugs, confirming that the simultaneous optimization of anti-resistance activity and toxicity can be achieved through structural modifications of the sugar side chain. However, this method is currently limited to a few known promiscuous glycosyltransferases. The majority of natural glycosyltransferases have extremely low activity with non-natural donors, thereby restricting the more general use of glycosyl exchange.

#### 3.1.3. Protein Engineering of Glycosyltransferases

The directed modification of glycosyltransferases through protein engineering can effectively overcome their highly specific natural substrate recognition to yield novel engineered enzymes with higher catalytic activity toward non-natural sugar donors with broader substrate compatibility [36]. Park et al. [37] constructed a library in which the domains of the kanamycin-derived glycosyltransferase KanM1 and the vancomycin-derived glycosyltransferase GtfE were randomly combined to create chimeric genes using a time-dependent truncation method. From this, a high-throughput plate screening system was established based on a pH indicator, which successfully generated a series of chimeric glycosyltransferases with excellent performance. This method did not require a complex enzyme structural analysis. It was simple to perform, highly efficient, and enabled glycosyltransferase substrate specificity to be precisely regulated and expanded. However, these enzyme evolution methods remain heavily constrained by the screening throughput, and their application scope is limited to targeting enzymes with suitable reporter systems.

These three paradigms—module replacement (AHBA side-chain assembly), element substitution (glycosyltransferase exchange), and enzyme engineering evolution (creation of chimeric glycosyltransferases)—together constitute the methodological framework for AG genetic circuit design and provide multiple technical routes for the creation of novel derivatives on a custom basis.

### 3.2. Chassis Cell Optimization

The optimization of chassis cells and the precise reconstruction of metabolic pathways are key steps in increasing the efficiency of natural product synthesis and producing structurally diverse antibiotics [38]. Ideal host strains should provide simple genetic manipulation, rapid growth, and a clear endogenous secondary metabolic background.

#### 3.2.1. Screening of Suitable Production Chassis Cells

*Streptomyces* and *Escherichia coli* (*E. coli*) are among the most widely used model hosts in current AG synthetic biology research [39]. *Streptomyces venezuelae* offers a well-established genetic manipulation system, stable growth characteristics, and a relatively simple endogenous secondary-metabolite profile. The absence of endogenous aminoglycoside biosynthesis minimizes pathway interference, making this species suitable as a heterologous expression chassis for complete AG BGCs [39]. Thapa et al. [39] first employed the aminoglycoside biosynthesis-deficient strain *Streptomyces venezuelae* YJ003 as a heterologous host and successfully achieved production of spectinomycin and its biosynthetic intermediates through expression of the spectinomycin BGC. This platform was shown to be suitable for the large-scale preparation of natural products and as a powerful tool for the analysis of biosynthetic pathways. Using this chassis, researchers successfully achieved the heterologous expression of the minimal gene sets of kanamycin and gentamicin A2 [24]. By leveraging the earlier work of Unwin et al. [40], in which the gentamicin C complex BGC was cloned and characterized, Park JW et al. [24] reassembled nine key genes dispersed in the gene cluster downstream of the erythromycin constitutive promoter PermE* to successfully produce gentamicin A2 in *Streptomyces venezuelae* YJ003. This work provided the first direct molecular genetic evidence for the complete biosynthetic pathway from G-6-P to the first trisaccharide intermediate, gentamicin A2. *E. coli*, with its well-characterized genetic background, simple molecular manipulation, and rapid growth rate, is more suitable than *Streptomyces* for the heterologous production of aminoglycoside precursors and core scaffolds, and for preliminarily functional validation of biosynthetic genes [10]. Consequently, additional regulatory elements and cofactor-supply pathways may be required to achieve efficient expression of complete aminoglycoside BGCs in this host.

#### 3.2.2. Genome Streamlining and Background Noise Reduction in Chassis Cells

Wild-type microbial genomes contain numerous nonessential genes, redundant secondary-metabolite BGCs, and restriction–modification elements that can consume cellular resources and interfere with the transcription and translation of heterologous genes [38]. Genome streamlining through the knockout of redundant genes, silencing of irrelevant endogenous secondary-metabolic gene clusters, and attenuation of host restriction–modification systems can simplify the chassis genetic background, reduce interference from endogenous gene expression, and improve the introduction efficiency and expression stability of heterologous BGCs without directly altering the AG biosynthetic pathway [24,40]. However, extensive genome reduction may impair host growth or increase sensitivity to fermentation conditions. Thus, effective chassis engineering requires a balance between genomic simplification and physiological robustness.

#### 3.2.3. Tolerance Characteristics of Chassis Cells and Fermentation Adaptation Optimization

AGs possess broad-spectrum antibacterial activities, and the continuous intracellular accumulation of antibacterial products can easily inhibit normal host growth and physiological metabolism. Therefore, enhancement of the tolerance of the chassis to aminoglycoside products and optimization of fermentation adaptation characteristics are important aspects of chassis engineering. By upregulating host self-resistance gene expression and optimizing extracellular transport systems, chassis cells can be adapted to tolerate high concentrations of aminoglycosides [39]. Adaptive laboratory evolution combined with fermentation optimization can further improve the growth stability, stress resistance, and high-density cultivation performance of the chassis host to meet both the needs of basic laboratory research and potential adaptation to industrial fermentation systems. Industrial strains, such as *M. echinospora* and *Streptomyces tenebrarius*, have been shown to possess good fermentation stability and product synthesis advantages after host adaptation engineering [41,42].

### 3.3. Cell-Free Synthesis

The scope of synthetic biology research and applications has long expanded beyond traditional living cell chassis factories [43]. In vitro enzymatic synthesis, also known as cell-free systems, is a flexible strategy for efficiently synthesizing and precisely modifying complex natural products that utilizes mild reaction conditions free from cellular toxicity constraints to enable simple product separation and purification and allow the reaction system to be precisely controlled [44].

#### 3.3.1. In Vitro Enzymatic Modifications Mediated by Inactivating Enzymes: Directed Derivatization

Aminoglycoside-inactivating enzymes typically possess remarkably broad substrate promiscuity and therefore provide a natural catalyst library for in vitro enzymatic modifications [6]. By using aminoglycoside acetyltransferases (AACs) and non-natural acyl-CoA analogs, the regioselective *N*-acylation of gentamicin, tobramycin, and other aminoglycosides was catalyzed in vitro to rapidly construct derivative libraries with novel biological activity [45]. Two of these AACs, AAC(6′)-APH(2″) and AAC(3)-IV, exhibited excellent substrate tolerance and efficiently catalyzed reactions involving various aminoglycosides and a range of acyl-CoA substrates [46]. This “reverse resistance” approach, which exploits bacterial resistance enzymes to create novel antibiotic structures, has strong potential for generating structural modifications of AGs.

#### 3.3.2. In Vitro Reconstitution of Biosynthetic Pathways: Mechanistic Elucidation

The ability to fully reconstitute biosynthetic pathways in vitro provides a powerful means of validating established pathways and uncovering cryptic mechanisms that may be difficult to resolve in complex in vivo environments. By completely reconstituting the gentamicin B biosynthetic pathway in vitro, researchers have identified three previously overlooked “cryptic” pathways and seven previously unrecognized intermediates. Kinetic analyses have further revealed that the substrate specificities of key glycosyltransferases and aminotransferases represent important factors limiting pathway productivity in vivo [28]. Notably, some newly identified minor components exhibit markedly reduced cytotoxicity in mammalian cells while retaining substantial nonsense mutation readthrough activity [47]. In vitro reconstitution has also demonstrated unique advantages in revealing distinctive enzymatic mechanisms, with the biosynthesis of the eight-carbon sugar core in apramycin being a classic example (see Section 4.5 for details) [48]. However, cell-free systems are currently constrained by the high cost of enzyme purification, low multi-enzyme cascade reaction efficiencies, and difficulties regenerating cofactors. Therefore, they are not yet ready for large-scale preparative applications.

### 3.4. Heterologous De Novo Biosynthesis

As understanding of AG biosynthetic mechanisms deepens, researchers have begun to move beyond the limitations of natural producers by constructing entirely new heterologous biosynthetic pathways de novo in non-producing microorganisms with well-characterized genetic backgrounds and rapid growth rates [49].

#### 3.4.1. Blockage of Competing Metabolic Branches

Natural aminoglycoside producers often have multiple coexisting components, with numerous byproducts that make separation and purification more difficult [43]. By precisely blocking competing metabolic branches through gene editing, the metabolic flux can been directed toward the target product for selective accumulation. For example, gentamicin C1a is an important synthetic precursor of etimicin [44]. Precisely knocking out the C-6′ methyltransferase gene *genK* in the gentamicin producer blocked the metabolic branches leading to gentamicin C2 and C1, and the yield of C1a increased nearly 10-fold [50,51]. Further knocking out of both *genK* and *genL* generated engineered strains that no longer produced the byproduct gentamicin C2b, as confirmed by mass spectrometry. Therefore, the strain specifically synthesized gentamicin C1a [52]. When this double-knockout method was applied to an industrial gentamicin-producing strain, the C1a yield increased by 3.22-fold [53], significantly improving the efficiency and cost-effectiveness of industrial production.

#### 3.4.2. Construction of Artificial Biosynthetic Pathways

Target products with extremely low natural yields can be efficiently produced through rational construction of artificial biosynthetic pathways. Gentamicin B is a key precursor in the preparation of the semisynthetic antibiotic isepamicin; however, its natural production yield is extremely low. To enhance gentamicin B yield, researchers first deleted the *genP* and *genK* genes in the producer strain, thereby effectively blocking competing downstream metabolic branches and redirecting the metabolic flux toward JI-20A accumulation [31]. Subsequently, heterologous expression the deaminase genes *kanJ* and *kanK* from the kanamycin biosynthetic pathway enabled the construction of an efficient artificial biosynthetic rote that converted gentamicin B from a trace product into the predominant product [54]. To further increase gentamicin B yield, the researchers overexpressed the glycosyltransferases KanM1 and GenM2 and initiated glucose feeding, increasing the yield by 54%, while the overexpression of KanM1 and GenM2 in a gentamicin C1a-producing strain increased the yield by 45% [55].

#### 3.4.3. Construction of Heterologous De Novo Biosynthesis

Heterologous de novo biosynthesis involves the stepwise assembly of complete biosynthetic gene modules in a non-native host and provides a powerful strategy for constructing AG biosynthetic pathways [56]. As a key precursor to the aminoglycoside core scaffold 2-DOS, 2-DOI represents an important starting point for heterologous de novo biosynthesis. In one study, the *btrC* gene from the butirosin biosynthetic pathway was introduced into *E. coli*, while key genes involved in competing G-6-P metabolic pathways were knocked out to reduce metabolic flux competition. This engineered system enabled efficient heterologous production of 2-DOI at a yield of 29.5 mg/L [57]. Building on the heterologous synthesis of 2-DOI, the 2-DOS biosynthetic genes *btrC*, *btrS*, and *btrN*, together with the glycosyltransferase gene *btrM,* were sequentially introduced into *E. coli,* enabling the successful biosynthesis of the aminoglycoside pseudodisaccharide scaffold paromamine at a yield of approximately 5 mg/L [58]. This study demonstrated that aminoglycoside core scaffolds need not be restricted to their natural *Streptomyces* producers and established that *E. coli* can reconstruct the 2-DOS-bsed aminoglycoside core scaffold de novo from simple carbon sources. However, the yields of heterologous de novo biosynthesis are orders of magnitude lower than those of natural producer strains, and the unbalanced expression of multiple rate-limiting enzymes in the pathway is a major bottleneck.

### 3.5. Systems Biology-Driven Global Optimization and Dynamic Regulation

The gene-editing and pathway-reconstruction strategies described above primarily focus on local targets, whereas efficient aminoglycoside production often requires coordinated optimization of the broader cellular metabolic network. Systems biology provides a genome-scale framework for characterizing and optimizing metabolic flux distribution, thereby addressing the multiple metabolic bottlenecks that are difficult to resolve through single-gene manipulation alone.

Using gentamicin C1a production, researchers constructed a GSMM of *M. echinospora*, designated as iFX1172, and predicted multiple pathway targets and global regulatory targets (*genC*, *metK*, *BldD*) through a flux balance analysis. After several modifications of these targets, the gentamicin C1a yield increased by 34.3% [13]. An AI-driven dynamic regulation system was further integrated into the process by combining backpropagation neural network modeling, non-dominated sorting genetic algorithm II for multi-objective optimization, dual-spectroscopy online monitoring, and closed-loop feedback control. Process parameters, including the substrate feed rate, pH, and dissolved oxygen, were modulated in real time according to the metabolic state of the cells during fermentation. This increased gentamicin C1a yield to 430.5 mg/L, a 75.7% improvement compared with conventional fed-batch fermentation systems [11,13]. A metabolic flux analysis demonstrated that this dynamic regulation reduced the glycolytic flux and tricarboxylic acid (TCA) cycle flux by 36.2% and 18.4%, respectively, while increasing the pentose phosphate pathway flux by 11.3%. The system also enhanced the nicotinamide adenine dinucleotide phosphate (NADPH) supply and increased the key precursor pools of glucose-1-phosphate and gentamicin X2 by 52.8% and 61.7%, respectively [59]. This case demonstrated that the integration of GSMM-guided target discovery and AI-assisted dynamic regulation systematically reshaped the cellular resource allocation and overcame global bottlenecks that traditional metabolic engineering cannot readily address. However, this type of system requires extremely high-quality multi-omics data and highly accurate computational models, and their applicability in actinomycetes is currently limited to only a few model strains.

### 3.6. Quantitative Comparison of Pathway Modification Strategies

To provide a more quantitative comparison of pathway modification strategies, representative examples are summarized in Table 2. These examples include metabolic branch blocking, heterologous de novo biosynthesis, glycosyltransferase swapping, and systems biology-guided dynamic regulation. For example, deletion of *genK* and *genL* increased the gentamicin C1a yield by 3.22-fold [53]; GSMM-guided modification combined with AI-driven dynamic regulation reached 430.5 mg/L C1a, a 75.7% increase compared with conventional fed-batch fermentation [11,13]; heterologous de novo biosynthesis in *E. coli* produced 29.5 mg/L of 2-DOI and approximately 5 mg/L of paromamine [57,58]; and glycosyltransferase swapping generated genkamicins at mg/L levels with reduced ototoxicity and nephrotoxicity [35]. These comparisons indicate that native-producer engineering currently can achieve higher titers, whereas heterologous de novo biosynthesis and hybrid derivative production remains limited by chassis compatibility, precursor supply, and enzyme compatibility. Direct comparisons should be interpreted with caution because strains, media, fermentation scales, and analytical methods differ among studies.

## 4. Distinct Pathway Features of Representative Aminoglycosides and Their Exploitation by Synthetic Biology

**Figure 4:**
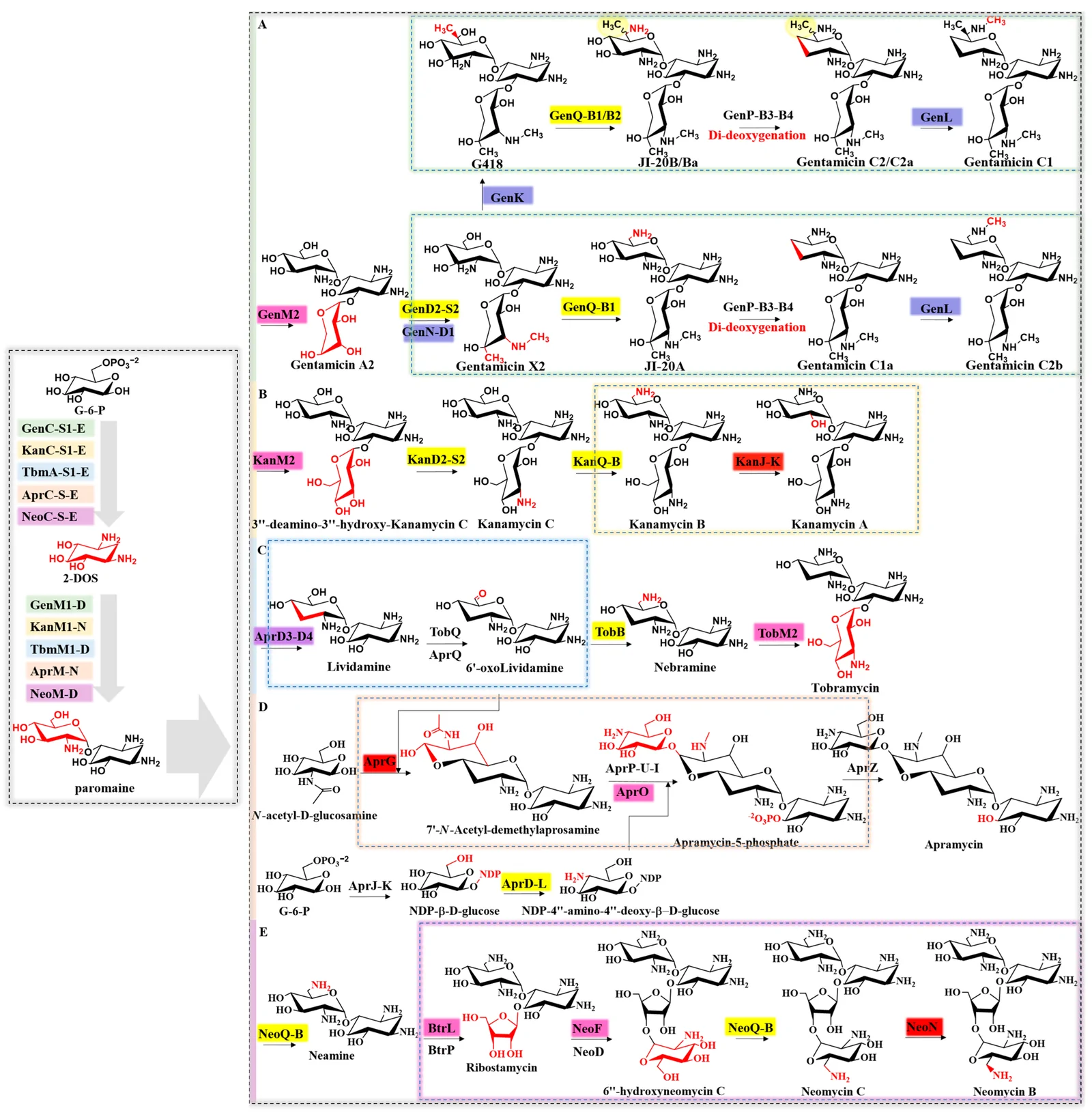
Distinct pathway features of representative aminoglycosides and their modifications by synthetic biology. (**A**) Gentamicin biosynthetic pathway. (**B**) Kanamycin biosynthetic pathway. (**C**) Tobramycin biosynthetic pathway. (**D**) Apramycin biosynthetic pathway. (**E**) Neomycin biosynthetic pathway. Chemical structures were drawn using ChemDraw.

**Figure 5:**
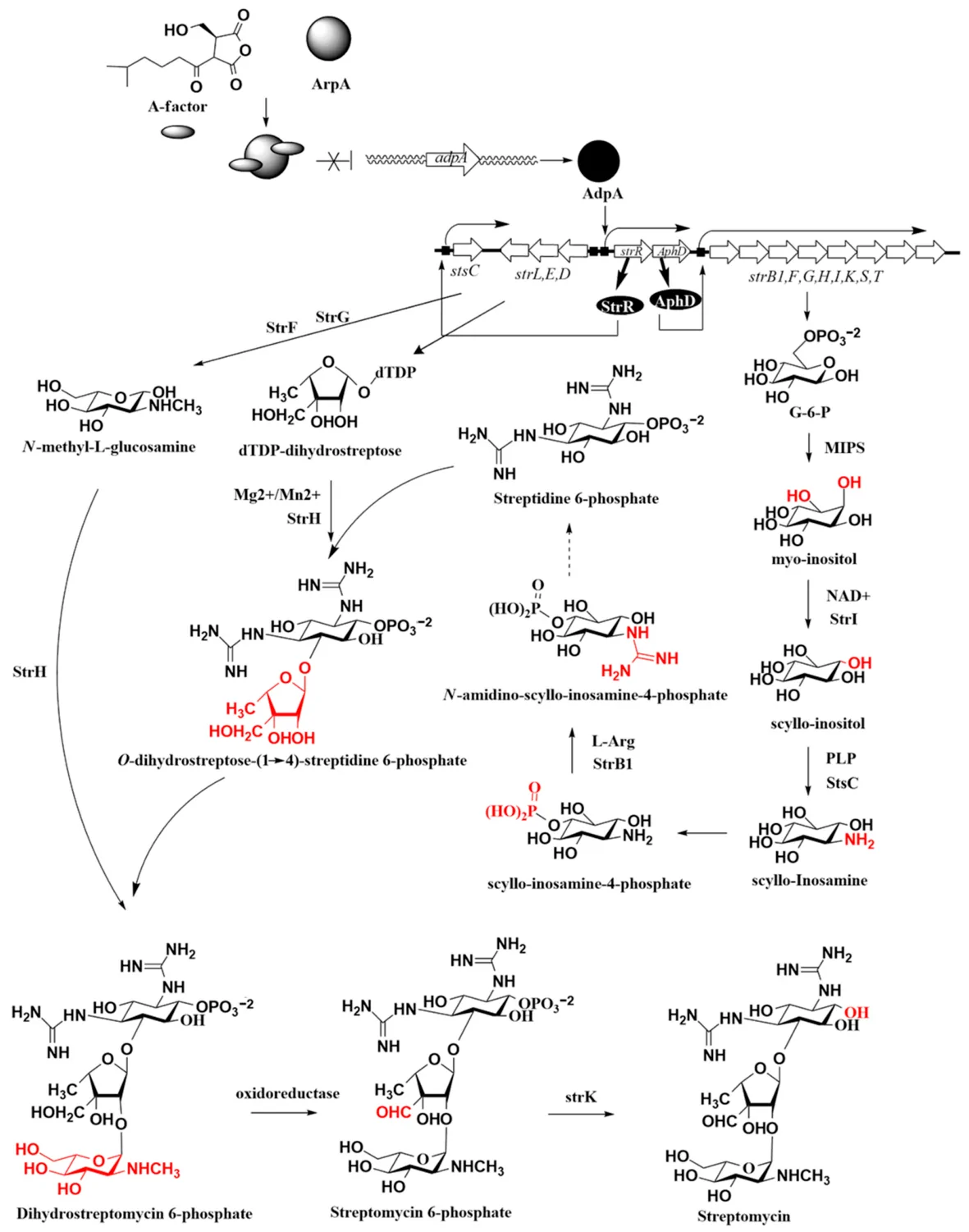
Biosynthetic pathway of streptomycin. Chemical structures were drawn using ChemDraw.

The biosynthesis of AGs is characterized by both evolutionary conservation and pathway diversity. Differences in the glycosyl chain assembly order, branching pathway architecture, regulatory mechanisms, and unique enzymatic steps among individual AGs provide diverse opportunities for synthetic biology-based engineering. Using gentamicin, kanamycin, tobramycin, apramycin, neomycin, and streptomycin as representative examples, this section discusses the key distinguishing features of their biosynthetic pathways and highlights synthetic biology strategies that can be leveraged to engineer these pathways. The major pathway features and engineering approaches are summarized in Figure 4 and Figure 5.

### 4.1. Gentamicin: Branching Network and Methylation Regulation Provide a Natural Target for Metabolic Branch Blocking

Gentamicin, produced by *Micromonospora* spp., is used clinically as a multicomponent mixture [61]. A distinctive feature of its biosynthesis is a complex branching network centered around the key branch-point intermediate gentamicin X2. Before X2 is synthesized, the glycosyltransferases GenM1 and GenM2 sequentially catalyze the attachment of UDP-*N*-acetylglucosamine and UDP-xylose to the C-4 and C-6 positions of 2-DOS, respectively, thereby completing assembly of the trisaccharide scaffold [62]. After X2 is synthesized, the pathway diverges into two parallel paths: X2 is methylated by GenK to generate G418 and subsequently JI-20B and JI-20Ba, while direct dehydrogenation and transamination yield JI-20A [9]. The C3′,4′-dideoxygenation and C5″ epimerization of terminal products further enrich gentamicin component diversity [29,30]. Notably, in this branching structure, single enzymes control multiple metabolic fluxes, with the presence or absence of GenK activity directly determining whether intermediates enter the G418 branch or the JI-20A branch. Additionally, substrate selectivity and mutual inhibitory relationships among the four methyltransferases GenN, GenD1, GenK, and GenL further refine the branching flux [9,63,64]. Therefore, knocking out or regulating only a few key enzyme genes can fundamentally redirect the product flux without the need for multi-target reconstruction of the entire pathway, making this pathway an ideal model for metabolic branch blocking strategies. As described in Section 3.4.1, precisely deleting *genK* and *genL* can block competing branches to direct nearly all metabolic flux toward C1a. After applying this approach to an industrial strain, the C1a yield increased by 3.22-fold and shifted the process from one producing a multicomponent mixture to single-component directed production [53,54]. In addition, the donor specificity of GenM2 for UDP-xylose provides an entry point for glycosyltransferase exchange techniques in which the glycosyl donor can be replaced to create structurally novel hybrid aminoglycosides (see Section 3.1.2 for details) [35].

### 4.2. Kanamycin: Linear Conversion Pathway and Component Ratio Regulation

Kanamycin is produced by *Streptomyces kanamyceticus* and primarily comprises two components, kanamycin A and kanamycin B, with kanamycin B exhibiting substantially greater antibacterial activity than kanamycin A [65,66]. Early studies proposed that the two components were synthesized independently through parallel pathways from a common precursor. However, Sucipto et al. [67] demonstrated that kanamycin A is generated from kanamycin B through C2’ oxidative deamination followed by reduction. This conversion is mediated by a coupled oxidative deamination–reduction module comprising KanJ, a kanamycin B dioxygenase, and KanK, an NADPH-dependent carbonyl reductase. Using in vivo gene knockout experiments, Gao et al. [60] confirmed that this linear conversion pathway represents the actual biosynthetic route in S. kanamyceticus, overturning the previously accepted model of parallel synthesis and establishing kanamycin B as the direct precursor of kanamycin A. Kharel et al. [66] subsequently cloned the kanamycin BGC and found that it is highly conserved, with the scaffold assembly region showing strong similarity to that of the gentamicin cluster, whereas the regions encoding late-stage tailoring enzymes differ markedly. This organization provides a molecular basis for the structural diversification of 2-DOS-containing aminoglycosides.

Because of this linear (B → A) conversion, the kanamycin pathway presents clear opportunities for metabolic engineering. The relative abundance of the two components is largely governed by the catalytic activities of KanJ and KanK. When kanamycin B is the desired product, suppression of KanJ and KanK can reduce downstream conversion and retain metabolic flux at the kanamycin B node. Gao et al. [60] demonstrated direct control of the component ratio and enrichment of kanamycin B by modulating KanJ and KanK expression levels. Thapa et al. [68] introduced the kanamycin BGC into *Streptomyces venezuelae* YJ003 for heterologous expression, thereby overcoming some genetic manipulation limitations of the natural host in genetic manipulation. Chemical deoxygenation strategies have expanded the structural diversity of kanamycin derivatives. For example, Yoneta et al. [69] synthesized 3′,4′-dideoxykanamycin A and 4′-deoxykanamycin A using dehydrated intermediates.

### 4.3. Tobramycin: The Deoxygenation Branch at the Disaccharide Stage

Tobramycin is produced by *Streptoalloteichus tenebrarius*. In wild-type *S. tenebrarius*, the radical SAM dehydratase complex AprD3–AprD4 exhibits strict natural substrate selectivity, preferentially recognizing the upstream intermediate paromamine and catalyzing its C3′ deoxygenation to yield lividamine. Lividamine is subsequently converted to nebramine through dehydrogenation and transamination catalyzed by TobQ and TobB, respectively, after which the glycosyltransferase TobM2 transfers UDP-xylose to complete the assembly of the trisaccharide scaffold, yielding tobramycin [70,71,72]. Metabolic flux analysis has shown that AprD3–AprD4 has substantially higher substrate affinity for paromamine than for neamine, resulting in inefficient entry of most intracellular neamine into the deoxygenation branch. In vitro, AprD3–AprD4 exhibits a broader substrate spectrum and directly catalyze C3′-deoxygenation of neamine to generate nebramine, which is subsequently glycosylated to yield tobramycin. This conversion route can only be realized in vitro in a reconstituted enzyme system, and it has an extremely low flux in wild-type strains [70,73]. The in vivo and in vitro deoxygenation pathways of tobramycin share a common feature: the C3′-deoxygenation modification occurs at the disaccharide intermediate stage, but not after the complete trisaccharide scaffold has been assembled [1].

This pathway architecture places the focus of tobramycin metabolic engineering on improving the efficiency of the deoxygenation module and optimizing metabolic flux through the nebramine branch. Feng et al. [74] enhanced tobramycin biosynthetic flux and reduced byproduct accumulation through metabolic pathway editing and overexpression of key enzymes in an industrial strain of *S. tenebrarius*. Shapovalova et al. [75] chemically modified the 6″-position of tobramycin, and the resulting derivatives exhibited enhanced antibacterial activity against resistant strains and reduced cytotoxicity relative to the parent compound. However, heterologous incorporation of the AprD3–AprD4 deoxygenation module for tobramycin production remains challenging because of the stringent substrate specificity of this enzyme complex and its dependence on a compatible redox environment.

### 4.4. Apramycin: [6 + 2] Condensation and Multi-Enzyme Synergy Underlie the Discovery and Application of a Novel Catalytic Element Library

Apramycin is produced by *S. tenebrarius*. It can evade the vast majority of AMEs and some 16S RMTases and has markedly lower ototoxicity than traditional 4,6-disubstituted aminoglycosides. Hence, it has received considerable renewed attention in recent years as a drug candidate against resistant bacteria [76]. Apramycin is the most structurally distinctive of the known aminoglycosides. In addition to 2-DOS and 4-amino-4-deoxyglucose, it consists of a unique bicyclic eight-carbon sugar core constructed through a [6 + 2] condensation reaction, constituting a rare example of naturally occurring carbon-carbon bond formation [48]. The apramycin pathway has three novel catalytic elements that can be recruited for use in synthetic biology.

#### 4.4.1. The Deoxygenation Module Composed of AprD3–AprD4

After synthesis of apramycin’s 2-DOS core, the sequential catalysis of the glycosyltransferase AprM and the deacetylase AprN generates paromamine. Subsequently, the radical SAM enzyme AprD4, which has a non-canonical CX_3_CX_3_C motif that binds a [4Fe-4S] cluster, catalyzes a dehydration reaction at the C3′ position of paromamine. The NADPH-dependent reductase AprD3 then reduces the dehydrated product to generate C3′-deoxylividamine, which is oxidized at the C6′ position by the dehydrogenase AprQ to yield 6′-oxolividamine [17,71]. This deoxygenation module was successfully applied in combinatorial biosynthesis with glycosyltransferases from the gentamicin and kanamycin pathways to construct hybrid aminoglycoside derivatives containing dideoxygenated structures [9,12].

#### 4.4.2. The [6 + 2] Condensation Reaction Catalyzed by the Transaldolase AprG

AprG catalyzes the cleavage of *N*-acetylglucosamine (GlcNAc) or *N*-acetylgalactosamine (GalNAc) to release an *N*-acetylethanolamine-type two-carbon unit. This unit then binds to the active center of AprG as a Schiff base intermediate and undergoes a stereospecific aldol addition with the aldehyde group of 6′-oxolividamine to form a new C–C bond. The addition product then undergoes intramolecular hemiacetal cyclization to construct a stable bicyclic eight-carbon skeleton, generating 7′-*N*-acetyldemethylaprosamine [48,77]. Kim et al. [77] recently elucidated the stereochemical inversion mechanism underlying this reaction through structural biology methods, providing a structural basis for engineering the substrate selectivity of AprG. Notably, AprG exhibits broad substrate tolerance and functions under mild reaction conditions, highlighting its potential as a versatile biocatalytic tool for constructing non-natural carbon skeletons. In particular, AprG may enable the synthesis of aminoglycoside analogs containing eight-carbon sugars or other atypical carbon frameworks, both in vitro and in heterologous hosts.

#### 4.4.3. The “Moonlighting” Glycosyltransferase Function of AprO, a Glycoside Hydrolase Family Member

After the bicyclic eight-carbon skeleton is assembled, AprJ (a phosphomutase) and AprK (a nucleotidyltransferase) activate G-6-P to NDP-β-D-glucose, and AprD (a dehydrogenase) and AprL (an aminotransferase) sequentially catalyze the C4 position oxidation and transamination to generate NDP-4″-amino-4″-deoxy-β-D-glucose. Finally, AprO, a glycoside hydrolase family member, acts as a glycosyltransferase to catalyze the linkage of this glycosyl donor to the C8′hydroxyl group of aprosamine, forming an 8′(e),1″(a)-*O*-glycosidic bond. After dephosphorylation by AprZ, the final product apramycin is generated [78]. This “one enzyme, multiple functions” catalytic approach is a new concept for the protein engineering of glycosyltransferases and raises the possibility that other glycoside hydrolases can also be “reprogrammed” as glycosyltransferases.

In one example of engineering and heterologous production, the apramycin gene cluster was heterologously expressed in *Streptomyces albus* and combined with CRISPR-Cas9-mediated gene editing to establish an efficient platform for constructing high-yield strains [17]. As the catalytic mechanism of AprG has been progressively elucidated and the substrate spectrum of the AprD3–AprD4 deoxygenation module has been expanded, coordinated integration of multiple catalytic modules offers new opportunities for aminoglycoside diversification. In particular, combining the AprG condensation, AprD3–AprD4 deoxygenation, and AprO glycosylation modules within a unified engineering framework may enable customizable biosynthesis of non-natural aminoglycoside derivatives containing eight-carbon sugars.

### 4.5. Neomycin: Linear Sequential Catalysis as an Ideal Model for Studying Glycosyl Chain Assembly

Neomycin is produced by *Streptomyces fradiae* in a biosynthetic sequence that follows a strictly linear sequential catalytic pattern. The reaction order cannot be reversed or skipped, and each glycosylation step is catalyzed by dedicated enzymes with extremely high substrate specificity [79,80]. This high degree of sequential dependence and enzyme specificity renders neomycin synthesis as an ideal model to research the sequential assembly logic of glycosyltransferases and glycosyl acceptor recognition. The entire pathway begins with glucose, with NeoC catalyzing the cyclization of G-6-P to generate 2-DOI. This is followed by transamination by NeoS and oxidative ring closure by NeoE to form the 2-DOS core scaffold [81,82]. NeoM and NeoD subsequently and sequentially catalyze the first glycosylation and deacetylation, respectively, to generate paromamine. NeoQ and NeoB then catalyze the dehydrogenation and transamination of paromamine to yield neamine. The phosphoribosyltransferase BtrL uses 5-phosphoribosyl-1-pyrophosphate as a glycosyl donor to convert neamine to 5″-phosphoribostamycin that is then dephosphorylated by the phosphatase BtrP to generate ribostamycin [80,83]. Ribostamycin is subsequently modified by the ribostamycin GlcNAc transferase NeoF, which transfers a GlcNAc group to ribostamycin. This is followed by deacetylation by NeoD to yield 6″-hydroxyneomycin C. NeoQ and NeoB subsequently catalyze dehydrogenation and transamination reactions, respectively, to produce neomycin C [79]. NeoN then catalyzes a configurational change in neomycin C at the C-5″ position, yielding the major active product neomycin B [84]. Because of this strict linear dependency, alteration of the order of any glycosyltransferase or replacement of its substrate specificity may disrupt the entire assembly line. To a certain extent, this limits the flexibility of achieving glycosyl diversification through single-enzyme replacement. However, it also provides a highly predictable framework for programmed design. As long as the recognition rules of each glycosyltransferase are individually analyzed, new glycosyl chain structures can be created by rearrangement or through the directed evolution of these enzymes.

NeoN catalyzes the stereochemical inversion at the C5 position of neomycin C to neomycin B. This is the most intensively studied radical SAM epimerase in aminoglycoside biosynthesis. Kudo et al. [84] elucidated the radical reaction mechanism of NeoN through in vitro enzymology and point mutation experiments. They showed that its [4Fe-4S] cluster, upon reduction, cleaved SAM to produce a 5′-deoxyadenosyl radical, which abstracted the hydrogen atom at the C5 position of the substrate to initiate epimerization. Clarification of this mechanism provided a structural foundation for subsequent directed evolution. Building on this, Li et al. [85] performed a semi-rational design of NeoN to obtain a V252A point mutant that possessed an enlarged substrate-binding pocket and increased the accumulation of neomycin B by approximately 40%. This successful case achieved the directed optimization of the neomycin component ratio and demonstrated that engineering of the terminal tailoring enzymes in linear pathways is an effective means of regulating the product spectrum.

The modular reconstruction of glycosylation was investigated by Fan et al. [79]. The deacetylase gene *neoD* was deleted, which demonstrated that its function could be partially compensated for by other esterases in the host. This revealed the presence of redundant deacetylase activity in *Streptomyces fradiae*. This finding indirectly illustrates that even in pathways with strict linear orders, certain auxiliary modification steps may still have a degree of substitutability, thus providing an entry point for pathway simplification and heterologous reconstruction. In recent years, the neomycin BGC has been heterologously expressed in model hosts such as *Streptomyces coelicolor*. In the future, by combining promoter engineering and ribosome binding site optimization, the neomycin pathway may be converted into a universal chassis for glycosyl chain assembly to customize non-natural glycosyl chain synthesis by replacing glycosyltransferase modules.

### 4.6. Streptomycin: A-Factor Cascade Regulation as a Portable Regulatory Element Library

Streptomycin, produced by *Streptomyces griseus*, was the first AG to be discovered. Its biosynthetic pathway is distinguished from those of other 2-DOS-containing aminoglycosides by two distinctive features: a transamidination modification involved in streptidine biosynthesis and the A-factor regulatory cascade. These features provide valuable sources of catalytic and regulatory elements for synthetic biology-based pathway engineering (Figure 5).

Streptidine biosynthesis is the most distinctive enzymatic module in the streptomycin pathway, and it follows a modular cascade of inositol → inosamine → transamidination. D-Glucose is phosphorylated by hexokinase to G-6-P, which is then cyclized and dephosphorylated by myo-inositol-1-phosphate synthase (MIPS) to generate myo-inositol [86]. Myo-inositol undergoes NAD^+^-dependent oxidation and epimerization to yield scyllo-inositol, and a PLP-dependent aminotransferase subsequently introduces an amino group at the C1 position to produce scyllo-inosamine [87]. The C4 position of scyllo-inosamine is then phosphorylated. This is followed by transamidination by transamidinase StrB1 using L-arginine as the amidino donor. The same pattern of oxidation → transamination → phosphorylation → transamidination is repeated at the C3 position, ultimately producing streptidine-6-phosphate [20]. The crystal structure of StrB1 has been determined, revealing that its five ββαβ modules are arranged in a five-fold symmetric ring that is topologically highly homologous to human L-arginine:glycine amidinotransferase [20]. This unique transamidination activity is unparalleled in aminoglycoside pathways. StrB1 serves as an amidination module in a catalytic element library, as it is theoretically capable of attaching amidino groups into other aminoglycoside scaffolds to alter their pharmacokinetic properties or evade recognition by resistance enzymes. Streptidine-6-phosphate is then coupled with dTDP-dihydrostreptose by the glycosyltransferase StrH to form *O*-α-L-dihydrostreptose-(1→4)-streptidine-6-phosphate [21]. This intermediate is subsequently assembled with *N*-methyl-L-glucosamine to generate dihydrostreptomycin-6-phosphate, which undergoes oxidation and phosphate hydrolysis by StrK to ultimately yield streptomycin [22] (Figure 5).

Transcription of the streptomycin gene cluster is tightly regulated by the A-factor signaling cascade, a classical paradigm of secondary metabolic regulation in actinomycetes. The A-factor (a γ-butyrolactone quorum-sensing signal molecule) binds to the receptor protein ArpA, which relieves the ArpA-mediated repression of the global regulator gene *adpA*. The AdpA protein is generated, which directly binds to the upstream activation sequence of the pathway-specific transcription factor gene *strR*, activating the transcription of *strR* and the entire downstream gene cluster [88,89,90]. Ohnishi et al. [89] overexpressed *adpA* to demonstrate that this regulator-enabled streptomycin synthesis begins at an earlier growth stage and also significantly increases the yield. This clear three-tier cascade amplification circuit (A-factor → ArpA → AdpA → StrR → structural genes) provides a portable regulatory element library for activating silent or weakly expressed secondary metabolic gene clusters in other actinomycetes.

Tomono et al. [90] placed *strR* under the control of a constitutive promoter and successfully restored streptomycin production in an *adpA* deletion mutant. This confirmed that structural genes were prematurely expressed under non-producing conditions bypassing A-factor cascade regulation. Therefore, the bottleneck in the streptidine precursor supply should be overcome by the heterologous expression of the complete streptomycin BGC in genetically tractable hosts, such as *Streptomyces lividans*, combined with metabolic flux analysis to enhance the inositol supply pathway [91].

## 5. Progress in the Development of Novel Derivatives

AGs face two long-standing challenges in clinical use: inherent ototoxicity and nephrotoxicity, and the continued emergence and spread of bacterial resistance. Traditional chemical semisynthesis has made limited progress in addressing these challenges [9,10]. However, recent advances in the understanding of aminoglycoside biosynthetic pathways and SARs, together with the rapid development of synthetic biology technologies, have established a new paradigm for the discovery and biosynthetic generation of novel aminoglycoside derivatives.

### 5.1. SARs: The Structural Basis of Activity and Toxicity

**Figure 6:**
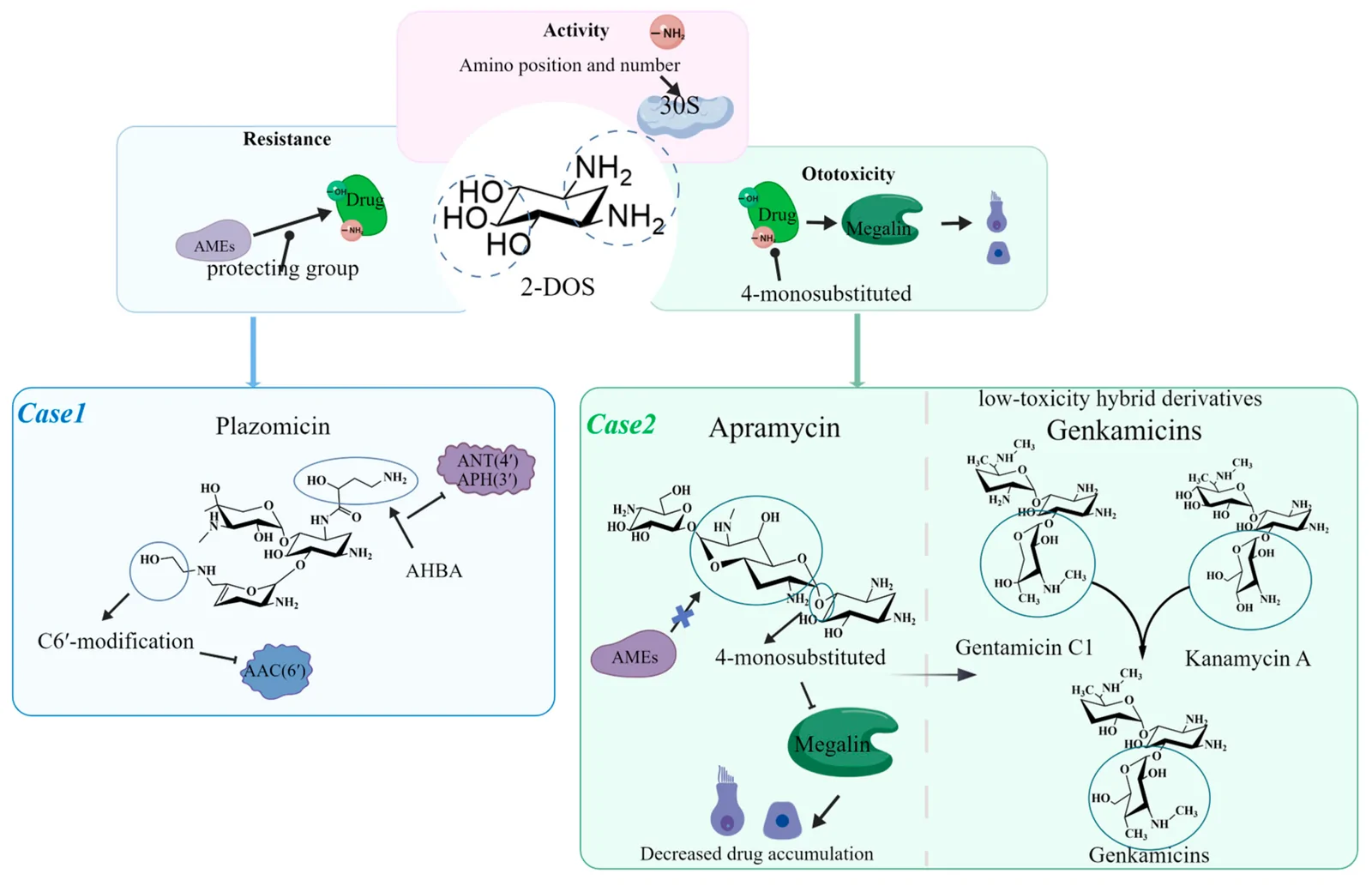
Structure–activity relationships of aminoglycosides and toxicity optimization strategies. Chemical structures were drawn using ChemDraw.

Understanding the SAR of AGs is essential for their rational design. Key structural determinants of aminoglycoside activity include the positions and number of amino groups on the 2-DOS ring, which directly influence the binding affinity for the decoding center of the bacterial 30S ribosomal subunit, as well as the specific hydroxyl and amino groups on the sugar rings that contribute to hydrogen bonding and electrostatic interactions with ribosomal RNA (rRNA) [3,92]. The resistance-related structural frameworks of AGs include key amino or hydroxyl groups of drug molecules that are recognized by AMEs for covalent modification. The introduction of protective groups at these sites (e.g., the AHBA side chain of amikacin) may effectively circumvent enzymatic modification [5,6]. The toxicity-related structural features underlying the ototoxicity and nephrotoxicity of aminoglycosides are closely related to the number and spatial arrangement of specific amino and hydroxyl groups in the different molecules [4]. Apramycin, a 4-monosubstituted 2-DOS-containing aminoglycoside, possesses markedly lower ototoxicity than commonly used clinical drugs such as neomycin and gentamicin. This indicates that the 4-monosubstituted structure significantly reduces the affinity of the drug for cochlear hair cells while retaining bacterial ribosome selective inhibition [62]. Megalin, a key transport receptor that accumulates aminoglycosides in renal tubular epithelial cells, has been confirmed to be closely associated with drug-induced nephrotoxicity and ototoxicity [92]. Furthermore, systemic inflammatory responses to infection can promote drug transport to the cochlea by upregulating transient receptor potential vanilloid 1 (TRPV1) channels and through other mechanisms, thereby markedly sensitizing ototoxicity [93]. These findings may provide molecular-level targets for designing low-toxicity derivatives (Figure 6).

The three-level SAR framework provides a clear molecular blueprint for the rational design of novel derivatives with respect to the two major challenges: addressing resistance, steric hindrance, or protective groups that can be introduced at AME recognition sites to block enzymatic modification; and addressing toxicity by reducing the affinity for megalin by decreasing the number of free amino and hydroxyl groups or altering the glycosyl substitution pattern. The following sections describe the progress made in developing derivatives based on SAR knowledge according to drug resistance and toxicity.

### 5.2. SARs in Resistance-Evading Design: The Success and Cautionary Lessons of Plazomicin

The primary mechanism of bacterial resistance to aminoglycosides is drug inactivation mediated by AMEs [5,6]. Additionally, target modifications mediated by 16S RMTases can cause high-level, broad-spectrum resistance [5,94]. Plazomicin is a third-generation AG produced by the rational structural modification of sisomicin. Its design logic was directly derived from a deep understanding of SARs and resistance mechanisms. An AHBA side chain introduced at the *N*1 position blocks recognition by multiple AMEs, such as ANT(4′) and APH(3′), and a 6′ position modification and 3- and 4′-hydroxyl group deletions further eliminates modification sites susceptible to enzymes such as AAC(6′) (Figure 6). Although plazomicin is prepared by chemical semisynthesis, its multi-site AME-blocking design is precisely what synthetic biology-based modifications attempt to realize systematically through the introduction of multiple protective modifications in one step through combinatorial biosynthesis in microbial cells. This drug was approved by the U.S. Food and Drug Administration (FDA) in 2018 for the treatment of complicated urinary tract infections. Plazomicin was the first novel aminoglycoside approved since amikacin, and it retains significant antibacterial activity against Enterobacteriaceae that produce extended-spectrum β-lactamases and carbapenemases [10].

However, success in resistance-evading designs is not permanent. Recent studies have identified a novel gentamicin resistance gene, *grdA*, carried by *Salmonella enterica*, that has been shown to mediate high-level resistance to plazomicin [6,95]. This is a fundamental dilemma in resistance-evading designs where even when multiple sites are protected against known AMEs, bacteria may still breach the defense by acquiring new enzyme-modifying genes. This finding indirectly shows that designs relying on a few static modification sites to block AMEs are inherently limited, whereas the ability of synthetic biology to dynamically generate diverse derivatives, such as rapidly creating structurally novel hybrid molecules through glycosyltransferase exchange, will be a key advantage in coping with the continuous evolution of antibiotic resistance.

### 5.3. Structural Insights for Low-Toxicity Design: The 4-Monosubstituted Template of Apramycin

The 4-monosubstituted structure of apramycin provides an important molecular template for low-toxicity designs. Matt et al. [62] systematically compared apramycin toxicity to classical 4,5-disubstituted (neomycin) and 4,6-disubstituted (gentamicin, tobramycin) aminoglycosides both in vivo and in vitro. They revealed that the 4-monosubstituted structure significantly reduced the affinity of the drug for cochlear hair cells while retaining bacterial ribosome selective inhibition. The molecular mechanism that reduces toxicity involved the following: first, under the 4-monosubstituted pattern, the overall charge density and the number of hydrogen-bond donors of the molecule were reduced, which lowered its affinity for megalin and thereby reduced drug accumulation in renal tubular epithelial cells and cochlear hair cells [92]; second, the unique bicyclic eight-carbon sugar core enabled apramycin to evade the vast majority of AMEs, thus avoiding the dose escalation and toxicity exacerbation caused by resistance (Figure 6). In a phase-I human clinical trial (NCT04105205) for the evaluation of the safety, tolerability and pharmacokinetics of single-dose apramycin [96], the antibiotic demonstrated promising research potential (study results not yet published) [97]. A second phase-I trial (NCT05590728) was subsequently conducted to evaluate its plasma pharmacokinetic (PK) distribution in epithelial lining fluid (ELF) and alveolar macrophages (AM), and no serious adverse events were observed.

Notably, apramycin is a natural product. Its low toxicity is the result of natural evolution rather than deliberate design. This demonstrates the value of synthetic biology to extract the low-toxicity design principle embodied by a 4-monosubstituted structure and apply it to the directed modification of other aminoglycoside scaffolds. This is precisely the logic underlying the creation of the hybrid genkamicin antibiotics described in the next section.

### 5.4. Low-Toxicity Hybrid Derivatives Created by Synthetic Biology: Genkamicins

Inspired by the 4-monosubstituted low-toxicity template of apramycin, researchers have further explored pathways that generate low-toxicity derivatives using synthetic biology. As described in Section 3.1.2, Jian et al. [35] created a series of structurally novel “gentamicin–kanamycin” hybrid aminoglycosides (genkamicins) through in vivo glycosyltransferase exchange. SAR and toxicity studies on the representative derivative GK-C2a showed that these hybrids retained potent antibacterial activity against resistant bacteria while exhibiting markedly improved ototoxicity and nephrotoxicity profiles [33,59]. In addition, the toxicity levels were found to be significantly lower than those of clinically used gentamicin and tobramycin in a zebrafish model (Figure 6).

Genkamicin development has methodological significance. First, it has been confirmed that the modification of the composition and linkage pattern of the sugar side chain can optimize toxicity without sacrificing antibacterial activity, providing a proof of concept for engineering glycosyl modifications to reduce toxicity. Second, it has demonstrated the unique advantage of synthetic biology in the development of derivatives, as it is challenging to use traditional chemical semisynthesis to achieve monosaccharide replacement at a specific position in a complex multicomponent system, such as that of gentamicin. Here, glycosyltransferase exchange only requires a single genetic manipulation to directly obtain the target hybrid product during fermentation. This marks a paradigm shift in aminoglycoside drug development from discovery in nature and chemical modifications to achieving antibiotic design and directed production within microorganisms.

### 5.5. From Laboratory to Industrial Production: Translational Bottlenecks and Future Directions

The maturation of combinatorial biosynthesis and metabolic engineering is driving aminoglycoside cell factories from proof-of-concept in the laboratory toward industrial use. The heterologous assembly of the AHBA side-chain module (see Section 3.1.1) and glycosyltransferase exchange (see Section 3.1.2) demonstrated the feasibility of the directed generation of non-natural derivatives in microbial cells. By integrating systems metabolic engineering and AI-assisted dynamic regulation (see Section 3.5), strains have achieved fermentation yields of single components such as gentamicin C1a that reach the threshold for industrialization [13,42,53]. For example, the shake flask yield of a sisomicin-specific production strain exceeded 1 g/L [42], indicating that synthetic biology-engineered strains now have the potential to compete with natural producers. However, the transition from laboratory to industrial fermentation still faces multiple obstacles. Heterologous expression yields are typically far lower than those of natural strains, the enzyme purification costs of cell-free synthesis systems are high, and the preclinical evaluation cycles for new derivatives are lengthy [98]. Currently, the core challenge in industrialization has shifted from whether a product can be synthesized to how the product can be synthesized economically, with the combination of cell-free synthesis systems and continuous flow processes expected to reduce production costs and overcome cellular toxicity limitations [7,15]. The early incorporation of techno-economic analyses and life cycle assessments will further help identify and prioritize the critical costs constraining commercialization.

The design directions for generating next-generation derivatives indicate that by integrating SAR knowledge with synthetic biology capabilities, next-generation aminoglycoside derivatives will be developed through an integrated design process encompassing three goals. In terms of resistance evasion, the multi-site protection design of plazomicin and the natural ability of apramycin will be drawn on to evade RMTases, and molecular scaffolds with immunity to both AMEs and RMTases should be designed. In terms of toxicity optimization, the 4-monosubstituted structure should be used as a template along with glycosyl exchange combined with glycosyl engineering to systematically explore the effects of different glycosyl combinations on megalin affinity and cochlear hair cell toxicity. In terms of efficient synthesis, the approach should shift from single chemical semisynthesis or single microbial fermentation to a hybrid manufacturing model that combines synthetic biology-created core scaffolds with in vitro enzymatic precision modification to balance structural diversity with production efficiency. With the continued discovery of new pathways using genome mining tools such as Seq2Saccharide [99] and the accelerated optimization of catalytic elements through AI-driven enzyme engineering, the development of aminoglycoside drugs is entering a new stage in which rational design, efficient synthesis, and safe use can be simultaneously achieved.

## 6. Challenges and Bottlenecks

Although synthetic biology has driven significant advances in AG research, several fundamental challenges remain unresolved across the continuum from biosynthetic pathway elucidation to industrial production. Further research is particularly needed in five interconnected areas: chassis adaptation, metabolic networks, resistance evolution, SARs, and industrialization.

### 6.1. Constraints on Heterologous Expression Chassis Adaptation

The transfer of BGCs from natural producers to model hosts is an attractive strategy to overcome genetic manipulation barriers, but chassis adaptation issues continue to hinder the practical implementation of this method [99]. Natural producer strains, such as *M. echinospora* and *S. tenebrarius*, have evolved highly specialized secondary metabolic regulatory networks and stringent precursor supply mechanisms over long evolutionary periods. Transferring these gene clusters to commonly used chassis such as *S. coelicolor* or *E. coli* frequently causes issues, including low transcriptional efficiency, inappropriate distribution of intermediate metabolic flux, target product instability, and even host toxicity [12]. Even with successful complete gene cluster transfer, heterologous expression yields are generally far lower than those of the original strains [100].

The root of this bottleneck is in the complex interactions between the primary metabolic networks of different chassis cells and the heterologous gene clusters. In addition, there is a lack of systematic strategies to guide the global metabolic adaptation engineering of the chassis [12,100]. Specifically, three factors jointly constrain the heterologous expression efficiency. First, promoters and ribosome-binding sites of actinomycete origin are often not effectively recognized in Gram-negative bacteria such as *E. coli*, which substantially reduces the transcriptional and translational efficiency. Second, cofactor supply networks that are coupled to secondary metabolism in natural producers (e.g., NADPH, SAM, and PLP) have markedly different capacities and regulatory patterns in heterologous chassis. This often leads to insufficient cofactor supply for key modifying enzymes. Third, aminoglycoside products themselves exert antibacterial activity against heterologous chassis, and even low-level leakage can inhibit host growth, forming a negative feedback loop. Overcoming these constraints requires the coordinated optimization of chassis transcription, translation, metabolism, and tolerance.

### 6.2. Multipoint Limitations of Metabolic Networks and Model Predictions

GSMM-guided combinatorial target intervention and AI-assisted dynamic regulation have achieved notable success, such as the engineering of gentamicin C1a; however, these successes have also exposed the limitations of current systems metabolic engineering approaches. Although GSMMs based on stoichiometric constraints can predict steady-state flux distributions reasonably well, they cannot capture dynamic constraints, such as enzyme kinetics, metabolite allosteric regulation, and post-translational modifications. In practical applications, experimental yields after a combination of model-predicted target modifications are often far lower than the theoretical optima. This indicates that numerous regulatory nodes in the metabolic network remain hidden in the models. Moreover, AI-driven dynamic regulation requires reliable online monitoring data, whereas the parameters available in real time in actinomycete fermentation systems are generally limited to pH, dissolved oxygen, and off-gas composition. Therefore, the real-time measurements of the substrate consumption rate and product formation rate still require offline sampling, which limits the response speed and precision of feedback control.

The poor transferability of multi-omics data across strains is another major obstacle. Each new strain requires the de novo collection of transcriptomic, metabolomic, and fluxomic data to train models, a lengthy and costly process. Furthermore, aminoglycoside biosynthesis involves deep coupling among multiple pathways, including glycolysis, the pentose phosphate pathway, the TCA cycle, and amino acid metabolism, and is not the simple superposition of a few rate-limiting enzymes. Therefore, local genetic manipulation may not be able to overcome multipoint limitations at the network level, as the metabolic network behaves like a buffering system replete with redundancy and compensatory circuits, with knockout or overexpression of a single node often absorbed by the overall elasticity of the network. GSMMs combined with AI may resolve this dilemma, but improving model prediction accuracy and establishing cross-strain transferability remain key determinants of whether this approach can be extended from individual cases to a general platform.

### 6.3. Development Challenges Posed by the Continued Evolution of Resistance

Plazomicin development, which systematically blocked the attack of known AMEs through multi-site protection, was once a landmark success in resistance-evading aminoglycoside design. However, the discouraging discovery of the *grdA* gene showed that even with systematic protection against multiple AMEs, such as APH(3′), AAC(6′), and ANT(4′), bacteria still restored resistance by acquiring novel modifying enzymes with different substrate spectra [6,95]. This case revealed a fundamental asymmetric competition in which researchers countered the known resistance mechanisms in a rational, target-by-target elimination manner, while bacteria rapidly acquired new defensive weapons in a combinatorial manner through horizontal gene transfer.

A core advantage of synthetic biology is its ability to rapidly generate diverse derivative libraries, which may be essential for reversing this asymmetry. Unlike chemical semisynthesis with its inherent modification site and yield limitations, synthetic biology can introduce multi-site modifications within microbial cells in one step through module recombination, thereby countering the speed of evolution with numbers. However, the entire process, from structural design to the identification of drug candidates, remains overly lengthy, and the linear workflow encompasses pathway reconstruction, product identification, activity screening, and toxicity evaluation that require up to months of work at each step. Shortening this cycle requires the establishment of a high-throughput “design–build–test–learn” closed loop to rapidly synthesize derivatives with parallel screening in cell-free synthesis systems. This enables the speed of iteration research and development to approach the evolutionary speed of resistance as closely as possible.

### 6.4. Insufficient Understanding of SARs and Lagging Toxicity Evaluation Systems

The clinical use of aminoglycosides is limited by their inherent ototoxicity and nephrotoxicity, while current SAR studies remain insufficient to support the rational design of low-toxicity derivatives [35]. Although the fundamental binding mode of aminoglycosides to the 30S ribosomal subunit has been established, three major gaps remain in current understanding.

First, the understanding of toxicity determinants is fragmented. Certain important aspects of toxicity and its attenuation are known: the 4-monosubstituted structure can significantly reduce ototoxicity, megalin is a key receptor for renal tubular accumulation, and inflammatory conditions sensitize cochlear toxicity through TRPV1 channels [62,92,93]. However, no systematic integrative framework has been developed to link these findings. Therefore, key aspects of toxicity remain unclear, such as how the same molecular structural features simultaneously affect ribosomal affinity, megalin binding, and mitochondrial toxicity. Second, there is a structural conflict between resistance evasion and toxicity optimization. Plazomicin blocks AMEs through an *N*1-AHBA side chain and multi-site modifications. However, these modifications increase the molecular weight and charge density, potentially increasing megalin binding and thereby increasing the nephrotoxicity risk. How to simultaneously optimize both resistance evasion and toxicity reduction through structural design remains a major challenge in SAR research. Third, the correlation between in vitro and in vivo toxicity is poor. Traditional in vitro cytotoxicity assays (e.g., HEK-293 cell lines) cannot faithfully reflect in vivo toxicity, in part because aminoglycoside toxicity causes long-term drug accumulation in cochlear endolymph and renal tubular epithelial cells, with enhanced transport under inflammatory conditions [93,101]. Zebrafish lateral line neuromast models and cochlear organoid models provide screening platform capabilities between those of in vitro systems and mammalian models, but their throughput and standardization are insufficient to meet the demands of drug screening.

### 6.5. The Translational Gap from Laboratory to Industrial Production

The synthetic biology-based engineering of AGs faces multiple obstacles in scaling up from laboratory to industrial production. These obstacles include technical, economic, and regulatory challenges.

The yield gap between heterologous expression and natural strains is the most direct technical bottleneck. Although metabolic branch blocking and systematic optimization of certain single components (e.g., C1a and sisomicin) have generated yields that approach or exceed 1 g/L [42,53], the heterologous yields of a majority of newly created hybrid derivatives (e.g., genkamicins) are at the mg/L level [35]. Although cell-free synthesis systems have demonstrated unique advantages in mechanistic studies and pathway validation, their industrialization remains challenging due to high enzyme purification costs, the time-dependent decay of multi-enzyme cascade reaction efficiencies, and the persistently high costs of cofactor regeneration (e.g., NADPH, SAM, ATP). CRISPR gene editing in actinomycetes is constrained by off-target effects associated with a high GC content, exogenous DNA degradation by restriction–modification systems, and the low efficiency of simultaneous multi-gene editing [102].

Economic and regulatory factors contribute to the lengthy and uncertain commercialization pathway of new derivatives. A majority of newly synthesized aminoglycoside derivatives cannot be converted into sufficiently useful compounds for commercialization [77]. An enormous translational gap exists between laboratory antibacterial activity data and meeting FDA/European Medicines Agency (EMA) clinical endpoints for complicated urinary tract infections or hospital-acquired pneumonia. In addition, aminoglycosides belong to an old drug class with relatively low market prices, and enterprises lack research and development motivation when the expected return on investment is minimal. Thus, this lack of economic need contributes to the slow advances in this field.

## 7. Conclusions and Prospects

The past two decades of research have shown that the study of AGs has undergone profound transformations, from discovery and description to design and creation. The driving force behind this transformations stem from a deep integration of synthetic biology and systems biotechnology, with synthetic biology providing engineering tools for reconstructing biosynthetic pathways and systems biotechnology providing a quantitative framework for understanding the global behavior of metabolic networks.

In terms of biosynthetic mechanisms, the assembly logic of the two core scaffolds, 2-DOS and streptidine, has been fully elucidated. Glycosyltransferases, PLP-dependent aminotransferases, SAM-dependent methyltransferases, and phosphorylation and deamination enzymes constitute a catalytic toolbox that is highly conserved in evolution and can be functionally exploited by engineering [11,14]. Although the enzymatic logic is conserved, the biosynthetic pathways of different antibiotics exhibit rich diversity, such as the branching network of gentamicin, the linear conversion of kanamycin, the [6 + 2] condensation of apramycin, the strict sequential assembly of neomycin, and the cascade regulation of streptomycin, each of which provides unique entry points for synthetic biology-based engineering [1,24,25,27,50]. Building on these engineering opportunities, a multi-layered engineering framework integrating genetic circuit design, chassis optimization, cell-free synthesis, heterologous de novo biosynthesis, and systems biology-driven global optimization has begun to emerge [13]. At the application level, genkamicin hybrid antibiotics generated through glycosyltransferase exchange have provided evidence that antibacterial activity can be partially decoupled from ototoxicity [27,35,103]. Additionally, the integration of metabolic branch blocking with AI-driven dynamic regulation has enabled high-yield production of individual aminoglycoside components [42,52,53]. The 4-monosubstituted structure of apramycin provides a portable natural template for designing low-toxicity derivatives [62]. Evidence from recent studies supports the feasibility of this approach. Glycosyltransferase exchange produced genkamicin derivatives with retained antibacterial activity and markedly reduced ototoxicity and nephrotoxicity [35]; the multi-site protection design of plazomicin demonstrated that resistance-evading scaffolds can be created by blocking AME recognition [10]; and the 4-monosubstituted scaffold of apramycin demonstrated that ototoxicity can be decoupled from antibacterial activity [62]. These examples indicate that synthetic biology can realistically contribute to overcoming resistance and toxicity, although further optimization and clinical validation remain necessary. Going forward, systematization, intelligentization, and green manufacturing are expected to become the three core drivers bringing this field to a higher level.

Systematization represents the direction of deepening cognition. GSMMs will need to evolve from steady-state stoichiometric descriptions toward dynamic models that integrate enzyme kinetics, allosteric regulation, and protein abundance information. Computational methods, such as transfer learning, are expected to lower the data threshold for constructing high-precision models of new strains and upgrading models from case-specific analytical tools to a universal platform deployable across strains. In addition, computational genome mining tools, such as Seq2Saccharide [99], are advancing natural product discovery from BGC identification to a new stage of pathway prediction and product structure prediction, thus providing computation-driven discovery engines to systematically explore novel aminoglycoside scaffolds.

Intelligentization is the catalyst for a leap in design capability. The application scope of AI is extending from fermentation parameter optimization to more central aspects. Algorithms based on protein language models and structure prediction to support enzyme engineering are expected to accelerate changes in the substrate selectivity of glycosyltransferases and methyltransferases and overcome the throughput bottleneck of directed evolution that relies on large-scale screening. Advances in pathway design, such as reinforcement learning methods, can search for optimal assembly schemes in vast module combination spaces to solve the combinatorial optimization problems of module selection and arrangement order when constructing multi-gene pathways. An ototoxicity and nephrotoxicity prediction framework that integrates molecular descriptors and physiologically based pharmacokinetic models may move safety assessments from late-stage development to the molecular design stage, with the potential to significantly reduce the late-stage failure rate of drug candidates.

Green manufacturing represents an innovation in production modes. By combining cell-free synthesis systems with continuous-flow bioprocesses, aminoglycoside production can shift from traditional batch fermentation toward continuous, modularized precision manufacturing. In a cell-free framework, the ratios of pathway enzymes and reaction conditions can be precisely programmed. Cellular toxicity limitations and competition from metabolic byproducts can be eliminated, making cell-free frameworks particularly suitable for the preparative synthesis of non-natural derivatives. CRISPR-mediated multi-gene editing and the iterative development of genome-streamlined chassis [102] will provide a standardized platform for rapidly constructing industrial strains, thus compressing the design-to-production cycle to several months.

These three drivers do not operate in isolation but mutually empower each other. Systematization provides a deep understanding of metabolic networks, intelligentization transforms this understanding into executable molecular designs and pathway schemes, and green manufacturing efficiently translates these schemes into scalable production processes. Through their coordination, the organic unification of resistance evasion, toxicity optimization, and innovation in synthesis methods will advance. As this paradigm progresses from concept to practice, the research and development of AGs is expected to enter a new stage of on-demand design, efficient synthesis, and safe use.

## Figures and Tables

**Table 1:** Comparison of representative previous reviews and the present review in terms of antibiotic classes, biosynthetic enzymes, engineering strategies, and emerging technologies.

Review	Antibiotic Classes	Biosynthetic Enzymes	Engineering Strategies	Emerging Technologies
Kudo & Eguchi [1] (2016)	2-DOS	2-DOS core scaffold biosynthetic enzymes	Focused on biosynthetic pathway elucidation; limited case studies and discussion	Not covered
Llewellyn & Spencer [2] (2006)	2-DOS	2-DOS core scaffold biosynthetic enzymes	Focused on biosynthetic pathway elucidation; limited case studies and discussion	Not covered
Ramirez & Tolmasky [6] (2010)	2-DOS	Not covered	Not covered	Not covered
Park et al. [11] (2017)	2-DOS	2-DOS core scaffold biosynthetic enzymes, glycosyl and tailoring enzymes	Combinatorial biosynthesis, heterologous biosynthesis	In vitro enzymatic reconstitution
Ban et al. [14] (2020)	2-DOS	2-DOS core scaffold biosynthetic enzymes and tailoring enzymes	Focused on modulating the biosynthesis of minor components and enrichment of low-abundance bioactive minor components	In vitro multi-enzyme pathway reconstitution
Rice et al. [15] (2025)	2-DOS	2-DOS core scaffold biosynthetic enzymes	Cell-free synthetic biology-based engineering strategies	Cell-free protein synthesis
Present review	2-DOS + streptidine	Core scaffold biosynthetic enzymes + four major classes of tailoring tool enzymes	Five strategies: Genetic circuit design (Section 3.1), Chassis cell optimization (Section 3.2), Cell-free synthesis (Section 3.3), Heterologous de novo biosynthesis (Section 3.4), and Systems-biology-driven global optimization (Section 3.5)	Genome-scale metabolic models, AI-assisted technologies, cell-free synthetic biology, genome mining tools, CRISPR-Cas9 multi-gene editing

2-DOS, 2-deoxystreptamine; AI, artificial intelligence. “Not covered” indicates that the item was not systematically addressed in that review.

**Table 2:** Quantitative comparison of representative pathway modification strategies for aminoglycoside biosynthesis and derivative production.

Target Product	Host/Chassis	Pathway Modification	Culture/Fermentation Condition	Quantitative Outcome	Ref.
Gentamicin C1a	*Micromonospora echinospora* industrial strain	Deletion of *gen*K and *gen*L to block competing branches	Industrial fermentation	C1a yield increased 3.22-fold	[53]
Gentamicin C1a	*M. echinospora*	GSMM-guided target modification + AI-driven dynamic regulation	Fed-batch fermentation	430.5 mg/L; 75.7% higher than conventional fed-batch fermentation	[11,13]
Gentamicin B	*M. echinospora*	Deletion of *gen*P and *gen*K; heterologous expression of *kan*J/*kan*K; overexpression of KanM1/GenM2 with glucose feeding	Fermentation	Yield increased by 54%, and by 45% in a C1a-producing strain	[54,55]
2-DOI	*Escherichia coli*	Expression of *btr*C; knockout of competing G-6-P metabolic pathways	Shake flask	29.5 mg/L	[57]
Paromamine	*E. coli*	Heterologous expression of *btr*C, *btr*S, *btr*N, and *btr*M	Shake flask	Approximately 5 mg/L	[58]
Sisomicin	Engineered strain	Pathway engineering	Shake flask	>1 g/L	[41]
Genkamicins	*M. echinospora*	Glycosyltransferase swapping using KanM2	Fermentation	mg/L level yield; reduced ototoxicity and nephrotoxicity	[35]
Kanamycin B/A ratio	*Streptomyces kanamyceticus*	Modulation of KanJ/KanK expression	Fermentation	Component ratio shifted toward kanamycin B	[60]

Direct comparisons should be interpreted with caution because strains, media, fermentation scales, and analytical methods differ among studies. C1a, gentamicin C1a; GSMM, genome-scale metabolic model; AI, artificial intelligence; 2-DOI, 2-deoxy-scyllo-inosose; G-6-P, glucose-6-phosphate.

## Data Availability

No new data were created or analyzed in this study. Data sharing is not applicable to this article.
